# Racial and weight discrimination associations with pain intensity and pain interference in an ethnically diverse sample of adults with obesity: a baseline analysis of the clustered randomized-controlled clinical trial the goals for eating and moving (GEM) study

**DOI:** 10.1186/s12889-021-12199-1

**Published:** 2021-12-02

**Authors:** Ericka N. Merriwether, Sandra Wittleder, Gawon Cho, Eushavia Bogan, Rachel Thomas, Naja Bostwick, Binhuan Wang, Joseph Ravenell, Melanie Jay

**Affiliations:** 1 New York University, New York, USA; 2 NYU Grossman School of Medicine, New York, USA; 3 New York Harbor VA, New York, USA

**Keywords:** Pain, Race, Racial discrimination, Weight, Weight discrimination, Sex, Gender, Weight management, Obesity

## Abstract

**Background:**

Everyday experiences with racial (RD) and weight discrimination (WD) are risk factors for chronic pain in ethnically diverse adults with obesity. However, the individual or combined effects of RD and WD on pain in adults with obesity is not well understood. There are gender differences and sexual dimorphisms in nociception and pain, but the effect of gender on relationships between RD, WD, and pain outcomes in ethnically diverse adults with obesity is unclear. Thus, the purposes of this study were to: 1) examine whether RD and WD are associated with pain intensity and interference, and 2) explore gender as a moderator of the associations between RD, WD, and pain.

**Methods:**

This is a baseline data analysis from a randomized, controlled clinical trial of a lifestyle weight-management intervention. Eligible participants were English or Spanish-speaking (ages 18–69 years) and had either a body mass index of ≥30 kg/m^2^ or ≥ 25 kg/m^2^ with weight-related comorbidity. RD and WD were measured using questions derived from the Experiences of Discrimination questionnaire (EOD). Pain interference and intensity were measured using the PROMIS 29 adult profile V2.1. Linear regression models were performed to determine the associations between WD, RD, gender, and pain outcomes.

**Results:**

Participants (*n =* 483) reported mild pain interference (T-score: 52.65 ± 10.29) and moderate pain intensity (4.23 ± 3.15). RD was more strongly associated with pain interference in women (*b* = .47, *SE* = .08, *p* < 001), compared to men (*b* = .14, *SE* = .07, *p* = .06). Also, there were no significant interaction effects between RD and gender on pain intensity, or between WD and gender on pain interference or pain intensity.

**Conclusions:**

Pain is highly prevalent in adults with obesity, and is impacted by the frequencies of experiences with RD and WD. Further, discrimination against adults with obesity and chronic pain could exacerbate existing racial disparities in pain and weight management. Asking ethnically diverse adults with obesity about their pain and their experiences of RD and WD could help clinicians make culturally informed assessment and intervention decisions that address barriers to pain relief and weight loss.

**Trial registration:**

NCT03006328

**Supplementary Information:**

The online version contains supplementary material available at 10.1186/s12889-021-12199-1.

## Background

Discrimination involves negative attitudes or unequal treatment towards individuals based on their characteristics (e.g., race, gender, weight status) [1–3]. Research suggests that frequent experiences with discrimination may be a stronger risk factor for pain among racialized groups than their White counterparts [1], potentially due to Non-Hispanic Black (NHB) and Hispanic/Latino/a/x adults’ increased exposure to multiple forms of discrimination. NHB and Hispanic/Latino/a/x adults may experience weight discrimination (WD) in combination with racial discrimination (RD) because obesity is highly prevalent among NHB and Hispanic/Latino/a/x adults [4]. Thus, it is critical to have a more refined understanding of the impact of discrimination on pain among individuals with obesity since pain is an established weight-related comorbidity [5–7]. Moreover, pain may accelerate health decline by functioning as a barrier to weight loss through increased sedentary behavior or through prolonged activation of regulatory hormones, cytokines, neuropeptides, and other secretagogues within the hypothalamic-pituitary-adrenal (HPA) axis [8–11].

Pain disparities associated with RD have been studied extensively. Frequent experiences with RD are associated with higher pain intensity [3], more pain-related disability [3], more bodily pain [12], and lower pain tolerance [13]. WD is also a prevalent form of discrimination in the U.S. [2, 14–16], and is associated with a wide variety of negative pain-related outcomes that include functional disability [17], psychiatric comorbidities [18], weight gain [19, 20], lower health-related quality of life [21], and higher mortality [22]. However, little is known about the impact of WD on pain-related outcomes in NHB and Hispanic/Latino/a/x adults with obesity who tend to be significantly underrepresented in pain studies.

Studies that have examined the burden and impact of multiple forms of discrimination on health suggest that characteristics that are perceived in society as ‘modifiable’ (e.g. weight) are likely to have a stronger negative effect on psychological outcomes associated with pain and weight management than discrimination based on ‘non-modifiable’ factors such as race and ethnicity [23]. Other studies have shown a dose-response relationship between multiple forms of discrimination and cardiovascular risk [24], and that WD, not RD, was significantly associated obesity risk [22]. These findings suggest that it is likely multiple forms of discrimination contribute to the burden of pain among NHB and Hispanic/Latino/a/x individuals. However, to our knowledge, previous studies have not partitioned the effects of RD and WD as risk factors for the development of chronic pain and disability.

Experiences with discrimination may differentially affect individuals that identify as women and men [1, 25]. Some evidence suggests that women are more likely to experience negative pain-related health consequences (e.g., psychological stress, anxiety, and pain catastrophizing) [23, 26] associated with discrimination, potentially due to the use of maladaptive coping strategies that may contribute to protracted activation of the HPA axis [9, 27]. Additionally, the social construction of female body ideals within a society may augment women’s and femmes’ vulnerability to experiences with WD, and thus, could exacerbate the negative impact of WD experiences on health [16, 28]. Also, gender differences in the association between discrimination and pain are vastly understudied in ethnically diverse populations with obesity, in part, due to an underrepresentation of men in pain, obesity, and weight management research [29, 30].

Thus, the primary aim of this study was to examine whether RD and WD are associated with pain intensity and interference in a racially and ethnically diverse sample of adults with obesity [31, 32]. Our secondary aim was to explore gender as a moderator of the effects of the associations between RD, WD, and pain. We hypothesized that more frequent experiences with the combination of RD and WD would be associated with higher pain intensity and greater pain interference. The objective of these analyses was to help identify novel factors that potentially contribute to disparities in pain-related outcomes in individuals with obesity.

## Methods

### Participants, study design, and setting

This is an analysis of baseline data from a randomized, controlled clinical trial (RCT) to test the efficacy of technology-assisted health coaching intervention for weight loss at two diverse urban healthcare systems in New York City: VA New York Harbor Healthcare System Manhattan Campus (VA) and four Montefiore Medical Group (MMG) primary care practices. The four MMG practices – Bronx East, Castle Hill, Grand Concourse, and University Avenue–are affiliated with the New York City Research and Improvement Networking Group. The trial was registered at clinicaltrials.gov (NCT03006328, December 30, 2016). The sample consisted of 483 primary care patients enrolled in this RCT. Study methods for the clinical trial are published in detail elsewhere [33]. All methods were performed in accordance with the relevant guidelines and regulations approved by the Institutional Review Boards at NYU School of Medicine (#i16–01445), VA NY Harbor (#01624), and Albert Einstein College of Medicine in collaboration with the Montefiore Health System (#2017–7603). All participants consented to study participation prior to data collection. The data were collected between 2017 and 2020. Baseline measurements were taken by research assistants during in-person visits to assess body measurements (e.g., height and weight), and to administer pain, discrimination, and other health surveys. Patients received 25 USD in compensation upon completion of the baseline data assessment.

#### Recruitment

We used electronic health records managed through the Veterans Health Information Systems and Technology Architecture (VistA), and Clinical Looking Glass™ (CLG), for the VA and MMG, respectively, to identify eligible patients. The majority of patients at the VA identify as male (90%) versus 48% across the MMG clinics. The patient populations at both sites are racially and ethnically diverse, with 21 to 55% of patients identifying as Hispanic/Latino/a/x and 37 to 53% as NHB. We sent out invitation letters and followed up with telephone calls to assess patients’ interest in study participation. If the patient expressed interest, we performed a telephone screen survey and conducted a chart review when necessary to determine study eligibility. Patients were then scheduled for the baseline visit.

#### Eligibility

Participants were primary care patients with either a body mass index (BMI) of ≥30 kg/m^2^ (obese) or ≥ 25 kg/m^2^ (overweight) with weight-related comorbidity (e.g., arthritis, sleep apnea, hypertension). Eligible participants were English or Spanish-speaking primary care patients between the ages of 18–69 years old who had at least one visit with their primary care provider in the past 24 months, access to a telephone, and the ability to travel for in-person visits. Patients with health conditions or those taking medications that could significantly affect weight change or impact their ability to participate were excluded. Some conditions that were part of the exclusion criteria were metastatic cancer, current chemotherapy or cancer treatment, diabetes, active psychosis, psychoactive substance use, Parkinson’s disease, or health problems that may prohibit the patient from participating in walking and/or physical activity such as chest tightness, a heart condition, or severe arthritis. Those taking weight loss and antipsychotic medications were also excluded from study participation [34]. We also excluded patients with a history of bariatric surgery or who were being evaluated for bariatric surgery, were pregnant, breastfeeding or planning to become pregnant during the intervention period, or participated in intensive weight management programs (> 4 sessions) in the past year. We also excluded patients who did not have self-reported ability to read English or Spanish at the 5th grade level and those with cognitive limitations that prevented them from adequately participating in a weight management program. Additionally, we did not enroll patients who were not interested in losing weight, as well as any patient whose primary care provider did not recommend study participation.

### Measures

#### Demographic characteristics

Data collected from the baseline questionnaire included information on participant demographics. The survey questions queried information on participants’ race and ethnicity, gender, civil service status, employment, highest level of education, and other demographic characteristics.

#### Racial discrimination (RD)

Participants completed the Experiences of Discrimination questionnaire (EOD) [35], which has been validated in patients with obesity and chronic pain [13, 24, 36, 37]. The EOD assesses the type and frequency of experiences with RD, and is comprised of questions that ask, “Have you ever experienced discrimination, been prevented from doing something, or been hassled or made to feel inferior in any of the following situations because of your race, ethnicity or color?” The frequency of nine discrimination experiences was assessed over a patient’s lifetime: “at school”; “getting hired”; “at work”; “getting housing“; “getting medical care“; “getting service at a store or restaurant“; “getting credit“, “bank loans or a mortgage“; “on the street or in a public place“; and “from the police or courts“. Available answer choices included: *never* (0), *yes, once* (1), *yes, 2–3 times* (2.5), and *yes, 4–5 times or more* (5). (Cronbach’s Alpha = .84). Survey responses were tallied to calculate a total summative score ranging from 0 to 45. Higher summative scores reflected more experiences with RD.

#### Weight discrimination (WD)

We assessed the type and frequency of experiences with WD using methods that have been previously validated in racially and ethnically diverse samples [18, 24, 28]. WD was assessed using a question from Wave 2 of the National Epidemiologic Survey on Alcohol and Related Conditions (NESARC), which is derived from the EOD survey [35]. Participants answered the question, “In the last 12 months, how often did you experience discrimination because of your weight?” along with the frequency of experiences with five modes of WD: “in your ability to obtain healthcare or health insurance coverage“; “in the way you were treated when you receive care“; “in public settings, like on the street“, “in restaurants or stores or on public transportation like buses or airplanes“; “in obtaining a job, or getting admitted to a school or training program“; and “in any other situation, like in the courts or by the police or when obtaining housing“. The response options were *Almost never* (1), *Sometimes* (2), *Fairly Often* (3), and *Very Often* (4). If participants responded *Sometimes* (2)*, Fairly Often* (3)*, or Very Often* (4) to any of the questions, we categorized them as having reported experiences with WD. We created a dichotomous variable coding: no reported WD (0) and reported WD (1) for each participant (Cronbach’s Alpha = .65). This categorization approach is consistent with previous studies [38].

#### Pain measurement

The pain interference and pain intensity subscales of the Patient-Reported Outcomes Measurement Information System (PROMIS) 29 adult profile V2.1 were used to assess pain. The PROMIS-29 has been widely used including in populations with obesity and chronic pain [39–45]. Participants answered four questions addressing pain interference (Cronbach’s Alpha = .94): “In the past 7 days, how much did pain interfere with your day to day activities”, “work around the house”, “your ability to participate in social activities”, “with your household chores”. The anchors on the subscale ranged from one to five, and were defined as: *not at all* (1)*, a little bit* (2)*, somewhat* (3)*, quite a bit* (4)*, very much* (5). We calculated a raw sum score with a minimum of 4 and a maximum of 20. Total raw scores were converted into a T-score, which has a mean of 50 and a standard deviation (SD) of 10 based on calibration testing performed on a large sample of the general population [46]. Thus, a person with a T-score of 40 is one *SD* below the average for the United States general population. Additionally, participants rated their pain intensity (“How would you rate your pain on average?”) on a scale from *no pain (0) to extreme pain* [10]*.* Summative raw scores were calculated and converted to T-scores in a manner similar to those previously described for the assessment of pain interference.

#### BMI

Body weight and height were measured at baseline. Participants’ BMI was calculated by dividing their weight in kilograms (kg) by the square of their height in meters (m), expressed as kg/m^2^. Participants’ body weight measurements were obtained via the HealthOMeter 349KLX Digital Medical Weight Scale using a standardized protocol which included weighing the participants without shoes or heavy garments. Body weight was measured twice to the nearest 0.10 pound (lbs.). If the difference between the two measurements was 0.50 lbs. or more, the measurement was repeated and the average of the two measurements that were closest in value were included in the analyses. Participants’ height was measured using the SECA 213 Portable Height, and the measurements were rounded up to the nearest 0.50 cm (cm). Participants were asked to modify hairstyles and remove their shoes as well as any extraneous clothing, if possible, to ensure measurement accuracy. Height was measured twice, and the average of the measurements was included in the analysis.

### Statistical analysis

Demographic characteristics were summarized using descriptive statistics. Mean and standard deviation were calculated for continuous variables, and frequencies and percentages were calculated for categorical variables. Participants were classified into four racial/ethnic groups using categories derived from the U.S. Census: Hispanic/Latino, Non-Hispanic White, Non-Hispanic Black (NHB), and Non-Hispanic Other. Differences in pain interference, pain intensity, and RD scores based on demographic characteristics were analyzed using the Mann–Whitney U test for dichotomous variables and the Kruskal-Wallis test for categorical variables with more than two levels. Differences in WD based on demographic characteristics were analyzed using the Chi-square test. Group differences in pain scores between WD groups (no reported WD (0) and reported WD (1)) were analyzed using the Mann–Whitney U test. Pairwise comparisons were conducted using the Bonferroni correction. The associations between pain interference, pain intensity, and RD scores were calculated using the Spearman’s rho correlation coefficient (r_s_). We used simultaneous linear regression models to examine independent relationships between WD, RD, and gender, with pain interference and pain intensity as the dependent variables. We also analyzed the interaction effects of WD, RD, and gender on pain intensity and interference in the model. We chose this model because we have no theoretical basis for considering any variable to be prior to any other. BMI, age, race, and enrollment site were included as covariates because they have been shown to be independently associated with RD, WD [47, 48], and with select pain outcomes [49, 50]. The main effects of RD, WD, and gender as well as two-way and three-way interaction effects were analyzed. If an interaction effect was significant, lower-order main and interaction effects nested beneath those analyses were not interpreted. Significant interactions were probed and plotted with the PROCESS macro for SPSS (version 3.04). A two-sided *p*-value < 0.05 was considered statistically significant, and all analyses were conducted using IBM SPSS Statistics for Windows, Version 25.0 (Armonk, NY: IBM Corp).

## Results

### Participant characteristics

Our sample was balanced in gender (56.3% female; 43.7% male) and the average age was 49.66 years (*SD* = 12.07). The average BMI was 34.83 kg/m2 (*SD* = 6.18), which falls in the obese range (BMI ≥ 30). Almost half of all participants identified as NHB (43.7%) and a little less than half of the participants (40.8%) reported their ethnicity as Hispanic/Latino/a/x. Pain interference had an average T-value of 52.65 (*SD* = 10.29) for the total sample. Mean pain intensity was 4.23 (*SD* = 3.15), and 59.1% of participants reported a pain intensity of 4 or higher on a scale from 0 to 10 for the total sample. Most participants worked full-time or part-time (62.7%). Approximately one-third (33.6%) of participants graduated from a 4-year college with 10% having earned a professional or graduate degree. Almost half (39.3%) of all patients were single/never married, and a similar number (36.2%) were married or in a long-term relationship (Table 1).

**Table 1:** Participant Characteristics in Pain Outcomes (Intensity and Interference), Racial Discrimination (RD), and Weight Discrimination (WD)

	Total *(n* = 483)	Pain Intensity	Pain Interference	RD	No WD	WD
**Enrollment Site**						
MMG	241 (49.9%)	4.00 (3.30)^1^	**50.98 (9.84)**^1^*	**4.91 (6.53)**^1^*	205 (85.1%)^4^	36 (14.9%)^4^
VA	242 (50.1%)	4.48 (2.97)^1^	**54.35 (10.46)**^1^*	**9.32 (9.93)**^1^*	194 (80.1%)^4^	48 (19.9%)^4^
**Gender**						
Women	273 (56.4%)	4.21 (3.22)^1^	52.11 (10.26)^1^	**5.79 (7.54)**^1^*	224 (82.3%)^4^	48 (17.7%)^4^
Men	211 (43.6%)	4.28 (3.05)^1^	53.40 (10.29)^1^	**8.81 (9.71)**^1^*	175 (82.9%)^4^	36 (17.1%)^4^
**Race/Ethnicity**						
Non-Hispanic Black	211 (43.6%)	4.17 (3.17)^2^	53.03 (10.44)^2^	**10.26 (10.07)**^**2,a,b**^	169 (80.5%)^4^	41 (19.5%)^4^
Non-Hispanic White	48 (9.9%)	4.08 (2.77)^2^	53.10 (10.83)^2^	**3.83 (5.66)**^**2,a**^	39 (81.2%)^4^	9 (18.8%)^4^
Non-Hispanic Other	26 (5.4%)	3.56 (3.03)^2^	51.27 (12.63)^2^	6.76 (8.29)^2^	22 (84.0%)^4^	4 (16.0%)^4^
Hispanic/Latino/a/x	197 (40.7%)	4.45 (3.21)^2^	52.39 (9.71)^2^	**4.66 (6.44)**^**2,b**^	167 (84.8%)^4^	30 (15.2%)^4^
**Employment Status**						
Working full-time	247 (51.0%)	3.92 (3.13)^2^	**51.05 (9.55)**^**2,a,b**^	6.62 (7.95)^2^	206 (83.7%)^4^	40 (16.3%)^4^
Working part-time	56 (11.6%)	3.95 (3.30)^2^	**50.19 (9.42)**^**2,c**^	4.49 (6.02)^2^	51 (91.1%)^4^	5 (8.9%)^4^
Unemployed or laid off/Looking for work	62 (12.7%)	4.92 (2.93)^2^	**55.41 (10.30)**^**2,b**^	8.83 (10.14)^2^	46 (74.8%)^4^	16 (25.2%)^4^
Student	15 (3.1%)	3.37 (2.91)^2^	53.59 (10.74)^2^	5.23 (6.96)^2^	12 (80.0%)^4^	3 (20.0%)^4^
Keeping house or raising children full time	13 (2.7%)	4.23 (4.02)^2^	51.60 (10.85)^2^	6.54 (9.66)^2^	10 (76.9%)^4^	3 (23.1%)^4^
Retired	91 (18.8%)	4.91 (3.02)^2^	**56.77 (11.20)**^**2,a,c**^	9.35 (10.42)^2^	74 (81.3%)^4^	17 (18.7%)^4^
**Education**						
Grades 5 through 11	20 (4.0%)	4.70 (3.67)^2^	51.48 (9.96)^2^	3.15 (5.72)^2^	16 (80.0%)^4^	4 (20.0%)^4^
Grade 12 or GED	112 (22.9%)	4.57 (3.38)^2^	52.93 (10.38)^2^	6.26 (9.35)^2^	89 (80.2%)^4^	22 (19.8%)^4^
Associates degree	58 (11.9%)	**3.07 (2.99)**^**2,a**^	51.14 (10.76)^2^	6.81 (6.86)^2^	52 (89.7%)^4^	6 (10.3%)^4^
Some college	133 (27.2%)	**4.69 (3.12)**^**2,a**^	53.39 (10.42)^2^	7.20 (8.03)^2^	109 (83.7%)^4^	21 (16.3%)^4^
College 4 years	102 (20.9%)	3.89 (2.85)^2^	52.72 (9.77)^2^	7.65 (9.29)^2^	83 (83.0%)^4^	17 (17.0%)^4^
Some graduate or professional training	12 (2.5%)	4.25 (3.08)^2^	53.51 (11.79)^2^	10.88 (9.89)^2^	7 (58.3%)^4^	5 (41.7%)^4^
Graduate or Professional degree	50 (10.2%)	4.28 (2.91)^2^	52.25 (10.31)^2^	8.63 (9.74)^2^	41 (82.0%)^4^	9 (18.0%)^4^
**Marital Status**						
Single/Never Married	190 (39.3%)	3.87 (3.23)^2^	52.18 (10.70)^2^	7.11 (7.83)^2^	153 (80.5%)^4^	37 (19.5%)^4^
Married or marriage-like relationship	175 (36.2%)	4.51 (3.09)^2^	53.01 (9.85)^2^	6.81 (9.38)^2^	152 (86.9%)^4^	23 (13.1%)^4^
Separated	23 (4.6%)	4.14 (3.09)^2^	51.45 (10.10)^2^	5.93 (8.29)^2^	18 (77.3%)^4^	5 (22.7%)^4^
Divorced	82 (17.0%)	4.23 (3.08)^2^	52.38 (10.02)^2^	7.42 (8.74)^2^	66 (81.5%)^4^	15 (18.5%)^4^
Widowed	13 (2.7%)	6.00 (2.65)^2^	58.57 (11.32)^2^	10.62 (11.21)^2^	9 (69.2%)^4^	4 (30.8%)^4^

*RD* Racial discrimination, *WD* Weight discrimination

Hispanic/Latino/a/x of any race including Black (*n* = 25), White/Caucasian (*n* = 26), None (*n* = 137); Multiple (*n* = 8), and Tainos Indian (*n* = 1). All other race categories are assumed to be non-Hispanic; Other include Asian (*n* = 5); American Indian/Alaskan Native (*n* = 1), Native-Hawaiian/Pacific Islander (*n* = 2), Multiple (*n* = 9), None (*n* = 6), West Indian (*n* = 2), and Middle Eastern (*n* = 1)

^1^Mann–Whitney U tests, ^2^Kruskal-Wallis tests, ^4^Chi-Square test

*p < .05 (two-tailed). Pairwise comparisons used Bonferroni correction. Means sharing a common subscript (a, a, a) are statistically different at *p* > .05

### Differences in pain interference and pain intensity

Participants enrolled at the VA NY Harbor site reported more pain interference than participants enrolled at MMG clinics. Patients who were unemployed or looking for work reported more pain interference than patients who worked full-time. Retired patients experienced more pain interference compared to patients who worked full or part-time. We did not observe differences in pain scores between gender, racial/ethnic groups, or marital status (Table 1). Additionally, there were no significant associations between pain interference, pain intensity, and age (Table S2).

### Differences in race and weight-based discrimination

NHB participants had EOD scores that were 2 times higher than the average scores reported by both Non-Hispanic White and Hispanic/Latino/a/x individuals (Table 1). Further, men-identifying participants’ average EOD scores were 1.52 times higher than women-identifying participants. Participants at the VA NY Harbor site reported more instances of RD than those at MMG clinics. We did not observe significant associations between RD and age (Table S2). Additionally, we did not observe differences in gender, racial/ethnic groups, employment, education (Table 1), or age (Table S3) between WD groups.

### Aim 1: characterize association between RD, WD, and pain

Findings from the linear regression analysis showed that more frequent experiences with RD were associated with more pain interference, (r_s_ = .27, *p* < .001) and higher pain intensity scores (r_s_ = .16, *p* = .001). Similarly, patients who experienced WD reported more pain interference, (*M* = 57.18, *SD* = 9.98 vs. *M* = 51.75, *SD* = 10.11), *Z* = -4.43, *p* < .001, and higher pain intensity scores, (*M* = 5.29, *SD* = 2.78 vs. *M* = 4.03, *SD* = 3.17), *Z* = -3.41, *p* = .001, compared with those who did not report WD (Table S3). After adjusting for BMI, age, and enrollment site, only RD predicted greater pain interference (Table 2), and higher pain intensity (Table 3). Contrary to our hypothesis, there was no significant interaction effect between RD and WD on pain interference or pain intensity (Tables 2 & 3).

**Table 2:** Pain Interference

	*Standardized Beta*	*SE*	*t*	*p*	*F*	*df*	*p*	*Adj. R2*
Model					6.01	12	<.001*	0.11
Gender^a^	0.06	1.39	0.96	0.34				
WD^f^	0.10	2.11	1.25	0.21				
**RD**	**0.46**	**0.11**	**4.89**	<.001*				
RDxWD	−0.14	0.12	−1.61	0.11				
**RDxGender**	**− 0.30**	**0.12**	**−3.30**	0.001*				
WDxGender	0.07	2.72	1.09	0.28				

^a^0 = female, 1 = male, ^b^0 = VA, 1 = MMG, ^c^0 = Non-Hispanic White, 1 = Non-Hispanic Black, ^d^0 = White, 1 = Hispanic/Latino/a/x, ^e^0 = White, 1 = Non-Hispanic Other, ^f^0 = no WD, 1 = WD

*p < .05

**Table 3:** Pain Intensity

	*Standardized Beta*	*SE*	*t*	*p*	*F*	*df*	*p*	*Adj. R2*
Model					2.95	12	.001*	0.05
Gender^a^	0.01	0.44	0.15	0.88				
WD^f^	0.06	0.67	0.73	0.47				
**RD**	**0.29**	**0.04**	**2.10**	0.004*				
RDxWD	−0.10	0.04	−1.06	0.29				
**RDxGender**	**−0.21**	**0.04**	**−2.20**	0.03*				
WDxGender	0.10	0.86	1.41	0.16				

^a^0 = female, 1 = male, ^b^0 = VA, 1 = MMG, ^c^0 = Non-Hispanic White, 1 = Non-Hispanic Black, ^d^0 = White, 1 = Hispanic/Latino/a/x, ^e^0 = White, 1 = Non-Hispanic Other, ^f^0 = no WD, 1 = WD

Age, race, and BMI were entered as covariates. For complete model statistics, see supplemental materials

*p < .05

### Aim 2: explore gender as a moderator of the association between RD, WD, and pain interference and intensity

**Fig. 1:**
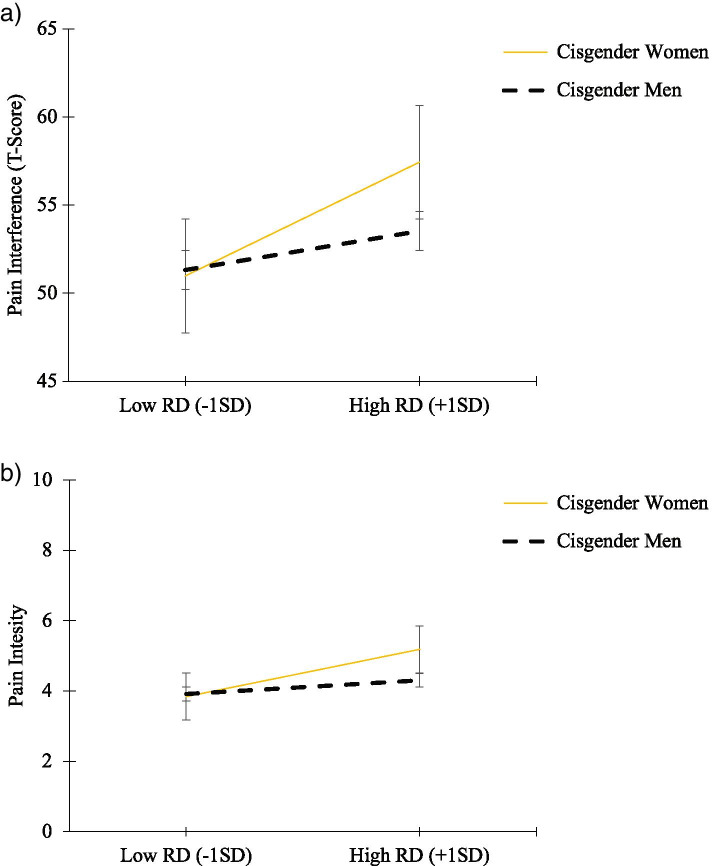
Pain interference scores (**a**) and pain intensity scores (**b**) by experiences of racial discrimination (RD) for self-identified gender groups. Note. Pain interference T-scores (**a**) and pain intensity scores (**b**) of participants who self-identified as male (cisgender men) or female (cisgender women) are shown for low (− 1 SD) and high (+ 1 SD) self-reported racial discrimination (RD)

The linear regression analysis revealed a significant interaction effect of RD X Gender on pain interference (Table 2). RD was significantly associated with pain interference in women-identifying participants (b = .47, SE = .08, CI[.303 to .629]) but not in men-identifying participants (b = .14, SE = .07, CI[−.005 to .282]) (Table 2, Fig. 1a). Similarly, RD was significantly associated with pain intensity among women-identifying participants (b = .08, SE = .03, *p* = .001 95% CI[.033 to .136]) but not in men-identifying participants (b = .03, SE = .02, *p* = 29 95% CI [−.021 to .070]) (Table 3, Fig. 1b). There were no significant interactions between WD X Gender on pain interference or pain intensity after adjusting for age, BMI, and enrollment site (Tables 2 & 3).

## Discussion

The current study characterized the relationships between experiences with two forms of discrimination (racial, weight) and pain outcomes, and examined if gender moderated these relationships. Results from this study confirm a high prevalence (59.1%) of at least moderate pain interference and pain intensity (4 out of 10 or higher) in adults with obesity. Also, experiences with racial discrimination (RD) are significant predictors of pain interference and pain intensity in a large sample of NHB and Hispanic/Latino/a/x adults with obesity having various chronic pain conditions. Further, gender identity moderates the association between pain interference, pain intensity, and RD. These results suggest that although men report more frequent experiences with RD, the associations between the frequency of experiences with RD, pain interference, and pain intensity are stronger in women, particularly in a large cohort of NHB and Hispanic/Latino/a/x adults with obesity. Another key finding is that participants who reported having experiences with weight discrimination (WD) had significantly greater pain interference, higher pain intensity, more experiences with RD, and a higher BMI compared to those who did not. However, WD was not a significant predictor of pain intensity or pain interference after statistical adjustment for experiences with RD, age, BMI, and enrollment site. As such, these findings suggest that RD and WD are experientially distinct phenomena, and thus, have a differential impact on the pain experience in adults with obesity. Surprisingly, there were no racial or gender differences in pain intensity or pain interference. To our knowledge, this is the first study that has investigated how gender moderates the relationships between RD, WD and pain outcomes in a large sample of racially and ethnically diverse adults with obesity.

Adults with obesity have a disproportionate burden of chronic pain. Approximately 75% of adults with obesity have chronic pain compared with 20.4% of the U.S. population [51]. Recent data in the United States have also shown that the age-adjusted prevalence of chronic pain is higher in women and military veterans [51]. Results from this study show a high prevalence of self-reported pain in a racially and ethnically diverse sample of participants - including military veterans - in a behavioral weight loss program, which is consistent with previous studies [52]. While NHB and Hispanic/Latino/a/x adults are known to have a disproportionately higher obesity prevalence [53], there were no significant differences in pain intensity or pain interference between racial groups in our study population. This contrasts with previous findings of higher self-reported pain in NHB, Hispanic/Latino/a/x, and Asian adults compared with NHW adults [54–58], though the results are inconsistent. Chronic pain prevalence is higher in NHB adults compared with NHW adults [59] in experimental [54, 55] and clinical [56, 60] settings. Further, Hispanic/Latino/a/x adults, particularly older adults, tend to have lower pain ratings and report less interference with functional activities compared with NHB and NHW adults [61–63]. Zettel-Watson et al. showed that 60% of Mexican-American older adults reported pain at multiple body sites, moderate to severe pain intensity, and that pain interfered with their normal work over the past 4 weeks [61]. Importantly, although pain is associated with health outcomes that are critical to the success of weight loss and is potentially influenced by metabolic or HPA axis dysfunction [9, 52], it is often unaddressed in weight management [64]. Our results highlight the need to query the severity and burden of pain so that pain interventions could be successfully incorporated into weight management. Moreover, given the paucity of pain research that is inclusive of NHB and Hispanic/Latino/a/x individuals with obesity, our findings suggest that experiences with RD and WD are salient and clinically relevant features of the pain experience in these populations, and the mechanisms underlying the relationships between discrimination and pain outcomes warrant further investigation.

Experiences with RD on NHB adults have deleterious effects on pain, obesity, and other health outcomes [1, 12, 13, 26, 37, 57, 63, 65, 66]. However, experiences with RD in Hispanic/Latino/a/x and other racialized groups are not well described. In previous studies, NHB adults reported more experiences of RD than Non-Hispanic White adults [1, 12, 13, 26, 57, 63], and more frequent experiences with RD were significantly associated with more pain interference and a higher pain intensity after adjusting for confounding variables. Altered nociceptive processing (e.g., heat pain tolerance), psychological factors, and sex/gender differences have been implicated as possible mechanisms underlying the relationship between RD and pain in NHB adults [26, 36, 54, 67–69]. Our findings show that NHB study participants reported more experiences with RD than Hispanic/Latino/a/x, Non-Hispanic White, or Non-Hispanic Other participants. These results suggest that NHB and Hispanic/Latino/a/x groups have different experiences with RD which, in turn, may give rise to disparate pain responses to it [61]. A potential reason for the differences in the reported instances of RD between NHB and Hispanic/Latino/a/x adults with obesity in the current study could be that Hispanic/Latino/a/x adults are not often specifically asked about the salient features of their experiences with RD such as language concordance, level of acculturation, and immigration status [70]. In previous studies, Hispanic/Latino/a/x adults have been asked about their experiences with racial or ethnic discrimination and its impact on pain in the context of access to primary care [71], provider bias [71], patient-provider language discordance, and immigration status [72]. Level of acculturation and assimilation into the dominant culture have also been cited as modes of discrimination by providers in a sample of Mexican-Americans [73]. Furthermore, the omnipresent fear of deportation - regardless of citizenship status - is significantly associated with pain-related outcomes, specifically stress and depression, as well as missed appointments for pain treatment [72]. These findings suggest a limitation in the way that questions about experiences with RD are asked to Hispanic/Latino/a/x adults. Thus, it is prudent to employ multimodal approaches to address the impact of RD on pain in NHB and Hispanic/Latino/a/x adults with obesity. Moreover, providers and researchers must consider asking culturally relevant questions and using surveys that specifically inquire about other features of RD (e.g., acculturation and language concordance) in ethnically diverse adults with obesity and chronic pain that have an immigrant experience in the United States.

Although sex and gender differences in the prevalence and trajectory of select chronic pain conditions have been well established [74–85], studies reporting gender differences in the relationships between RD and pain outcomes in ethnically diverse pain populations have been sparse and inconclusive. In a robust sample of primary care patients with chronic musculoskeletal pain, women-identifying participants reported more pain interference than men-identifying participants [86]. The majority of the cohort of women-identifying participants (54%) were NHB women. Terry et al. found that despite reporting more experiences with RD, there were no significant relationships between RD and pain in NHB older men with knee osteoarthritis [26]. Conversely, in a sample of NHB older men (military veterans), RD was a significant predictor of bodily pain [12]. Notably, NHB and Hispanic/Latino/a/x men with obesity are frequently underrepresented in pain studies, so sex and gender differences in pain interference and experiences with RD are particularly not well understood in these patient populations. The current study shows that the associations between RD, pain interference, and pain intensity are stronger in women-identifying participants with obesity compared to men-identifying participants - the majority of whom identify as NHB and Hispanic/Latino/a/x. Previous findings from a large, multi-ethnic cohort show significant correlative relationships between experiences with RD and bodily pain in Japanese, Chinese, African American, Caucasian, and Hispanic women, but they did not compare their experiences to those of men [63]. In the same study, NHB women also reported having more frequent experiences with RD whereas Hispanic/Latino/a/x women reported the lowest frequency of RD experiences [63]. However, Hispanic/Latino/a/x women had the highest pain ratings at baseline compared with NHB, NHW, Japanese, Chinese women [51]. Dugan et al. posited that other forms of discrimination, particularly related to gender and English fluency, could have also been captured by the EOD in their study cohort though not directly assessed [63].

Some purported mechanisms for the gender differences in the relationships between experiences with RD and pain interference are related to differences in affective dimensions of pain such as coping, pain self-efficacy, and pain beliefs [26, 74, 75, 87–95]. Pain catastrophizing, a cluster of negative emotions related to magnification, rumination, and helplessness around pain [96], and perceived stress have been found to moderate the association between discrimination, pain intensity, and pain interference in women when demographic variables are controlled [26]. Although stress was not measured in this investigation, women-identifying participants may have experienced stress more intensely than their male counterparts, which could explain the stronger association between RD and pain interference in women despite reporting less frequent experiences with RD than men-identifying participants [26]. Stress alleviation is a key component of weight and pain management given the shared pathophysiology of prolonged elevation of cortisol, a steroid hormone, in obesity and chronic pain [9]. Surprisingly, there were no race or gender differences in pain interference in our sample population. However, the impact of adiposity and body image are under recognized forms of discrimination that may influence pain chronicity, and should be addressed in pain and weight loss interventions.

WD is increasingly recognized as a social determinant of health. WD and RD have been identified as the most common forms of repeated daily discrimination in racially and ethnically diverse populations of adults with obesity [97]. Importantly, WD is associated with increases in BMI, and weight gain [19]. The current study shows that participants who identify as NHB or Hispanic/Latino/a/x were able to disentangle their experiences with WD from their experiences with RD. Gee et al. reported similar findings in a large cohort of Asian ethnic groups in the context of increased BMI [98]. The group found that WD was significantly associated with increased BMI. Further, the associations between RD and BMI were significant when controlling for the influence of WD. Of note, the majority of participants in the sample were not classified as having obesity using World Health Organization (WHO) criteria (< 10%). Other researchers have reported that NHB participants cited body appearance, in addition to RD, as a potential mechanism for the amplification of their experiences with discrimination [1, 2]. In the current study, WD was not associated with pain outcomes after statistical adjustment though participants that reported having experiences with WD had significantly higher pain intensity, greater pain interference, and more experiences with RD. A potential reason for these discrepant findings is that the number of participants reporting experiences with WD were underrepresented in the total study population (< 25%). Thus, we may have been underpowered to analyze the contribution of WD to the variance in pain interference and intensity. Mehok and colleagues suggest that patients’ weight and gender identity influence observers’ perceptions of pain severity, the rate of referral for physical therapy services, and recommendations to engage in physical activity as an adjuvant therapy for pain control [99].

### Limitations

There were some limitations associated with the study. First, this is a secondary data analysis with the primary study outcome being weight loss. Furthermore, study participants were not recruited based on the presence or absence of a regional or widespread musculoskeletal pain condition. However, our findings may be more generalizable to adult populations with obesity. Moreover, the robust battery of health surveys and questionnaires that queried pain and discrimination afforded us the opportunity to analyze these relationships in understudied populations. Secondly, pain surveys and questionnaires can be prone to recall biases, and we did not administer clinical or experimental dynamic pain modulatory assessments. Lastly, we did not assess anticipatory or enacted discrimination which could have different relationships to pain intensity or pain interference.

## Conclusion

Findings from this study demonstrate that pain is prevalent in adults with obesity, specifically racially and ethnically diverse adults enrolled in a comprehensive behavioral weight management program. Further, we have expanded on results from previous studies by characterizing racial and gender differences in the experiences with RD and WD in a robust sample of ethnically diverse adults with obesity. Asking patients about their experiences with discrimination based on race, ethnicity, or weight could help clinicians make culturally informed decisions about ways to assess pain and the selection of interventions in adults with obesity that maximize pain relief and weight loss. Future studies should build on these findings by investigating whether training providers to ask about and validate experiences of RD and WD has prognostic and therapeutic benefits. Additionally, clinicians and researchers could collaboratively develop and clinically validate intervention strategies that account for frequent experiences with RD and WD.

## Supplementary Information

**Additional file 1: Table S1.** Frequencies of participants’ gender by race/ethnicity. **Table S2.** Descriptive Statistics and Spearman’s rho Correlations for Study Variables. **Table S3.** Results Of Mann–Whitney U tests To Investigate Differences Between Patients Who Experienced WD And Who Did Not Experience WD. **Table S4.** Pain Interference. **Table S5.** Pain Intensity.

## Data Availability

The datasets and materials used and/or analyzed during the current study available from the corresponding author on reasonable request.
