# Supplementary material for: Racial and weight discrimination associations with pain intensity and pain interference in an ethnically diverse sample of adults with obesity: a baseline analysis of the clustered randomized-controlled clinical trial the goals for eating and moving (GEM) study
Source: BMC Public Health. 2021 Dec 2;21:2201. doi: 10.1186/s12889-021-12199-1 (PMC8638106; doi:10.1186/s12889-021-12199-1)
Supplement: Supplementary file 1 — Additional file 1: Table S1. Frequencies of participants’ gender by race/ethnicity. Table S2. Descriptive Statistics and Spearman’s rho Correlations for Study Variables. Table S3. Results Of Mann–Whitney U tests To Investigate Differences Between Patients Who Experienced WD And Who Did Not Experience WD. Table S4. Pain Interference. Table S5. Pain Intensity. [file 12889_2021_12199_MOESM1_ESM.docx]

Supplemental

Table S1

| Frequencies of participants’ gender by race/ethnicity | | | |
| --- | --- | --- | --- |
|  | Female  n(%) | Male  n(%) |  |
| Non-Hispanic Black | 125 (59.24) | 86 (40.76) |  |
| Hispanic/Latino/a/x | 122 (61.93) | 75 (38.07) |  |
| Non-Hispanic White | 11 (22.92) | 37 (77.08) |  |
| Non-Hispanic Other | 13 (50.00) | 13 (50.00) |  |
| Note. | | | |

**Table S2**

| *Descriptive Statistics and Spearman’s rho Correlations for Study Variables* | | | | | | |
| --- | --- | --- | --- | --- | --- | --- |
| Variable | *n* | *Mean* (*SD*) | 1 | 2 | 3 | 4 |
| 1. Pain Interference, T-score | 483 | 52.65 (10.29) | - |  |  |  |
| 2. Pain Intensity | 482 | 4.23 (3.15) | .71*** | - |  |  |
| 3. RD | 479 | 7.09 (8.67) | .27*** | .15** | - |  |
| 4. BMI, kg/m^2^ | 484 | 34.83(6.18) | .09* | .11* | .07 | - |
| 5. Age, years | 483 | 49.66 (12.07) | .08 | .08 | .05 | -.10* |
| *Note.* BMI = body mass index, RD = racial discrimination  **p*<.05*;* ***p*<.01, ****p*<.001 | | | | | | |

**Table S3**

| *Results Of Mann–Whitney U tests To Investigate Differences Between Patients Who Experienced WD And Who Did Not Experience WD* | | | | | |
| --- | --- | --- | --- | --- | --- |
|  |  | No WD  *n*=399 | WD  *n*=84 |  |  |
|  | *n* | *M*(*SD*) | *M*(*SD*) | *Z* | *p* |
| 1. Pain Interference, T-score | 482 | 51.75 (10.11) | 57.18 (9.98) | -4.43 | **<.001** |
| 2. Pain Intensity | 481 | 4.03 (3.17) | 5.29 (2.78) | -3.41 | **.001** |
| 3. RD | 478 | 5.55 (7.06) | 14.59 (11.46) | -7.33 | **<.001** |
| 4. BMI, kg/m^2^ | 483 | 33.91 (8.03) | 39.29 (8.03) | -6.20 | **<.001** |
| 5. Age, years | 483 | 49.96 (12.04) | 48.36 (12.07) | -1.18 | .24 |
| *Note.* BMI = body mass index, RD = perceived racial discrimination, WD = weight discrimination | | | | | |

**Table S4**

*Pain Interference*

|  | *Standardized Beta* | *SE* | *t* | *p* | *F* | *df* | *p* | *Adj. R2* |
| --- | --- | --- | --- | --- | --- | --- | --- | --- |
| **Model** |  |  |  |  | **6.01** | **12** | **<.001*** | **0.11** |
| **BMI** | **0.11** | **0.08** | **2.33** | **0.02*** |  |  |  |  |
| **Age** | **0.10** | **0.04** | **2.26** | **0.03*** |  |  |  |  |
| Gender^a^ | 0.06 | 1.39 | 0.96 | 0.34 |  |  |  |  |
| **Enrollment Site^b^** | **-0.14** | **1.16** | **-2.56** | **0.01*** |  |  |  |  |
| Non-Hispanic Black^c^ | -0.05 | 1.70 | -0.59 | 0.56 |  |  |  |  |
| Hispanic/Latino/a/x^d^ | 0.04 | 1.67 | 0.53 | 0.60 |  |  |  |  |
| Non-Hispanic Other^e^ | -0.01 | 2.41 | -0.24 | 0.82 |  |  |  |  |
| WD^f^ | 0.10 | 2.11 | 1.25 | 0.21 |  |  |  |  |
| **RD** | **0.46** | **0.11** | **4.89** | **<.001*** |  |  |  |  |
| RDxWD | -0.14 | 0.12 | -1.61 | 0.11 |  |  |  |  |
| **RDxGender** | **-0.30** | **0.12** | **-3.30** | **0.001*** |  |  |  |  |
| WDxGender | 0.07 | 2.72 | 1.09 | 0.28 |  |  |  |  |

*Note*.

^a^ 0 = female, 1 = male, ^b^0 = VA, 1 = MMG, ^c^0 = Non-Hispanic White, 1 = Non-Hispanic Black, ^d^0 = White, 1 = Hispanic/Latino/a/x, ^e^0 = White, 1 = Non-Hispanic Other, ^f^0 = no WD, 1 = WD.

**p*<.05*;* ***p*<.01, ****p*<.001

**Table S5**

*Pain Intensity*

|  | *Standardized Beta* | *SE* | *t* | *p* | *F* | *df* | *p* | *Adj. R2* |
| --- | --- | --- | --- | --- | --- | --- | --- | --- |
| **Model** |  |  |  |  | **2.95** | **12** | **.001*** | **0.05** |
| **BMI** | **0.12** | **0.03** | **1.90** | **0.06*** |  |  |  |  |
| **Age** | **0.12** | **0.01** | **2.63** | **0.01*** |  |  |  |  |
| Gender^a^ | 0.01 | 0.44 | 0.15 | 0.88 |  |  |  |  |
| Enrollment Site^b^ | -0.09 | 0.37 | -1.56 | 0.12 |  |  |  |  |
| Non-Hispanic Black^c^ | -0.01 | 0.54 | -0.16 | 0.87 |  |  |  |  |
| Hispanic/Latino/a/x^d^ | 0.11 | 0.53 | 1.33 | 0.19 |  |  |  |  |
| Non-Hispanic Other^e^ | -0.01 | 0.76 | -0.12 | 0.90 |  |  |  |  |
| WD^f^ | 0.06 | 0.67 | 0.73 | 0.47 |  |  |  |  |
| **RD** | **0.29** | **0.04** | **2.10** | **0.004*** |  |  |  |  |
| RDxWD | -0.10 | 0.04 | -1.06 | 0.29 |  |  |  |  |
| **RDxGender** | **-0.21** | **0.04** | **-2.20** | **0.03*** |  |  |  |  |
| WDxGender | 0.10 | 0.86 | 1.41 | 0.16 |  |  |  |  |

*Note*.

^a^ 0 = female, 1 = male, ^b^0 = VA, 1 = MMG, ^c^0 = Non-Hispanic White, 1 = Non-Hispanic Black, ^d^0 = White, 1 = Hispanic/Latino/a/x, ^e^0 = White, 1 = Non-Hispanic Other, ^f^0 = no WD, 1 = WD.

**p*<.05*;* ***p*<.01, ****p*<.001
